# Antibiotics Resistance in *Escherichia coli* Isolated from Livestock in the Emirate of Abu Dhabi, UAE, 2014–2019

**DOI:** 10.1155/2022/3411560

**Published:** 2022-04-26

**Authors:** Ghada Elderdiri Abdelwahab, Hassan Zackaria Ali Ishag, Zulaikha Mohamed Al Hammadi, Saeed Mohamed S Al Yammahi, Mohd Faoruk Bin Mohd Yusof, Muna Sayed Y. Al Yassi, Shaikha Saeed A. Al neyadi, Asma Mohammed A. Al Mansoori, Fawzia Hassan A. Al Hamadi, Ibtesam Abdullah S. Al Hamadi, Mohamed Ali Abdalla Al Hosani, Salama Suhail Mohammed Al Muhairi

**Affiliations:** Veterinary Laboratories Division, Animal Wealth Sector, Abu Dhabi Agriculture and Food Safety Authority (ADAFSA), Abu Dhabi, UAE

## Abstract

*Escherichia coli* (*E. coli*) is a zoonotic pathogen that showed growing resistance to antibiotics. No descriptive analysis highlights the threat of antimicrobial-resistant (AMR) of *E. coli* among livestock in the United Arab Emirates (UAE). Herein, we conducted phenotypic and genotypic resistance studies on *E. coli* isolates from livestock samples in the Emirate of Abu Dhabi based on routine diagnosis between the periods 2014–2019. Bacterial culture and disk diffusion methods were used for bacterial isolation and phenotypic resistance analysis. Resistance mechanism was studied by PCR targeting the most commonly resistance genes: ampicillin (*bla*_*SHV*_, *bla*_*CMY*_, and *blaTEM-1B*), tetracyclines (*tetA* and *tetB)*, co-trimoxazole [sulfamethoxazole (*sul1*, *sul2*, and *sul3*) + trimethoprim (*dfrA1* and *dfrA17)*], aminoglycosides [*aph(3”)-Ia*, *aph(6)-Id,* and aac(3)-IV], and fluoroquinolones (*qnrA* and *aac(6')-Ib-cr*). Analysis of 165 *E. coli* isolates showed resistant to ampicillin, tetracycline, co-trimoxazole, gentamicin, and enrofloxacin by 157/165 (95.4%), 154/165 (93.6%), 141/165 (86%), 139/165 (85%), and 135/165 (82.7%), respectively. Predominant resistance gene/s detected by PCR were *bla*_*CMY*_ (119/160, 72%) and *blaTEM-1B* (154/160, 96.3%) for ampicillin; *tetA* (162/164, 98.8%) and *tetB* (112/164, 68.3%) for tetracyclines; *sul2* (156/164, 95%), *sul3* (138/164, 84%), and *dfra17* (74/164, 44.5%) for co-trimoxazole; *aph(3”)-Ia* (134/164, 82.1%) and *aph(6)-Id* (161/164, 98.2%) for aminoglycosides; and *aac(6')-Ib-cr* (61/61, 100%) for enrofloxacin. Both phenotypic and genotypic analyses revealed that all *E. coli* isolates were multidrug-resistant (resistance to 3, 4, and 5 antibiotics classes by 3.6%, 57.6%, and 38.8%, respectively) carrying one or more resistance gene/s for the same antibiotic. PCR profiling confirmed the presence of resistance genes corresponding to their antibiotic profile. Results of the study will highlight the knowledge based on *E. coli* AMR related to livestock in UAE that may call for interventions.

## 1. Introduction

*Escherichia coli* (*E. coli*) is a Gram-negative rod bacterium taxonomized within the family of Enterobacteriaceae. *E. coli* is a ubiquitous bacterium isolated from humans, animals, birds, and different environments. In humans and animals, *E. coli* normally exists in the intestine as microflora and plays an important role in digestion and absorption [1]. On the contrary, *E. coli* can be pathogenic or zoonotic, causing serious diseases such as diarrhea, hemorrhagic colitis (HC), and hemolytic uremic syndrome (HUS) in both humans and animals [2, 3].

Antimicrobials are normally used in livestock to treat diseases caused by *E. coli*, such as diarrhea, gastrointestinal infections, mastitis, and urinary tract infection (UTI). Nevertheless, the uses of antimicrobials in the treatment of livestock or as a growth promoter have been linked to the development of multidrug-resistant *E. coli*, a threat to public health [4–6]. Indeed, *E. coli* isolated from livestock samples has been reported to show different resistances to several antimicrobials, including erythromycin, tetracycline, ampicillin, gentamicin, sulfamethoxazole/trimethoprim, chloramphenicol, kanamycin, and streptomycin [7–13]. This resistance phenomenon is also an economic burden for farmers because of costs incurred in treatment failure and a prolonged period of treatment of the bacterial infections [14]. In UAE, the common antibiotics used to treat animals include tetracycline, oxytetracycline, penicillin, ampicillin, amoxicillin, oxacillin and cefoxitin, gentamicin, sulfamethoxazole/trimethoprim (co-trimoxazole), enrofloxacin, and ceftiofur. However, the current knowledge based on *E. coli* AMR against these antibiotics concerning livestock in UAE is unclear.

The detection of antimicrobial resistance was mostly done phenotypically assessing the sensitivity to these antibiotics as recommended by the Clinical and Laboratory Standards Institute (CLSI) and/or the European Committee on Antimicrobial Susceptibility Testing (EUCAST) [15]. The molecular detection based on polymerase chain reaction (PCR) has been widely used to detect the genes responsible for the resistance against several antibiotics [11].

Antimicrobial-resistant bacteria have been detected in a variety of domestic animals and environments [16, 17]. Studies from the Middle East highlight the spread of resistant organisms in hospitals and to a lesser extent in livestock and the environment [18]. In UAE, most of AMR studies in *E. coli* are related to humans [19–21]. Yet, *E. coli*-based AMR surveillance in animals in UAE is lacking.

Based on the samples that were analyzed and diagnosed in our laboratories for bacterial infection, we found a considered number of *E. coli* isolates showed resistance to several antibiotics commonly used for animal treatment by the veterinarians in Abu Dhabi Agriculture and Food Safety Authority (ADAFSA), Abu Dhabi, UAE. Therefore, we aimed to study the AMR in *E. coli* isolated from the Emirate of Abu Dhabi livestock to understand the resistance level in the region that may pose a risk to public health. We applied the antibiotic susceptibility test and PCR to screen the antibiotic resistance in *E. coli* and explore the potential resistance mechanisms. To our knowledge, this is the first comprehensive study that provides useful information for veterinary practices and public health concerns in UAE.

## 2. Materials and Methods

### 2.1. Sampling, Bacterial Isolation, and Identification

A total of 165 samples from different regions of the Emirate of Abu Dhabi [Al Ain = 108/165 (65.5%), Al Dhafra = 20/165 (12.1%), and Abu Dhabi = 37/165 (22.4%) samples] were received by our laboratory from January 2014 to May 2019 for bacterial infection investigation. The samples include organs (liver, kidney, heart, lungs, spleen, intestine, and lymph nodes), blood, milk, urine, pus, uterine discharges, and fecal swabs from different animals (camel = 42/165 (25.5%), sheep = 42/165 (25.5%), goat = 54/165 (32.7%), and poultry = 27/165 (16.4%)). The animal samples cultured onto a range of general and selective bacterial culture media (purchased from Pharmatrade, Dubai, UAE), including blood agar (incubated aerobically and anaerobically), MacConkey, Xylose Lysine Deoxycholate agar (XLD), Eosin methylene blue (EMB), Brilliant Green Agar (BG) and nutrient agar (NA). The cultured media were incubated at 37°C for 24 hours aerobically and 48 hours anaerobically. Bacterial identification was done using the VITEK II identification system (bioMérieux). For this study, each isolate was harvested into a microbial cryopreservation beads system (Mast Cryobank) according to the manufacturer's instruction and stored at −80°C.

A written consent, which was included in the sample request form, approved by ADAFSA research ethics committee for the use of the samples for publication, was obtained from the animal's owners before inclusion in the study.

### 2.2. Antimicrobial Susceptibility Testing

Antimicrobial susceptibility was performed against the most commonly used antibiotics for animal treatment in UAE tetracycline, oxytetracycline, penicillin, ampicillin, amoxicillin, oxacillin and cefoxitin, gentamicin, co-trimoxazole, enrofloxacin, and ceftiofur using disk diffusion method according to the guidelines of Clinical and Laboratory Standards Institute (CLSI-VET01 5^th^ Edition/VET08 4^th^ Edition). A panel of five antibiotics disks was used, which include, ampicillin (10 *μ*g), enrofloxacin (5 *μ*g), gentamicin (10 *μ*g), tetracycline (30 *μ*g), and co-trimoxazole (25 *μ*g) and a combination of trimethoprim and sulfamethoxazole 1.25/23.75 *μ*g antibiotics.

### 2.3. Detection of AMR Genes by PCR in E. coli-Resistant Isolates

#### 2.3.1. DNA Extraction

From each cryovial (representing one isolate), a single bead was removed aseptically with a sterile needle, streaked over a plate of blood agar, and incubated overnight at 37°C. Then, for genomic DNA extraction, 3–5 isolated colonies were suspended in 200 *μ*L phosphate-buffered saline (PBS) for manual extraction with QIAamp® DNA Mini Kit (Qiagen) following the manufacturer's instructions. The DNA was quantified by Nanodrop 2000 (Thermo Fisher Scientific, USA) and used as a template for PCR.

#### 2.3.2. Primers and PCR Analysis

Based on antibiotic-resistant phenotype of *E. coli* and previous studies, we selected common resistant gene/s per antibiotic to be tested by PCR [11, 12, 22]. These genes were as follows: ampicillin (*bla*_*SHV*_, *bla*_*CMY*_, and *blaTEM-1B*), tetracycline (*tetA* and *tetB)*, trimethoprim (*dfrA1* and *dfra17*), sulfamethoxazole (*sul1*, *sul2*, and *sul3*), gentamicin [*aac(3)-IV*, *aph(3”)-Ia*, and *aph(6)-Id*], and fluoroquinolones (*qnrA* and *aac(6')-Ib-cr*).

The primers used the genes (*bla*_*SHV*_, *bla*_*CMY,*_*tetA, tetB, dfrA1, sul1, aac(3)-IV*, and *qnrA*) were previously described [11, 23–28]. Other genes including *blaTEM-1B, dfra17, sul2*, *sul3*, *aph(3”)-Ia*, *aph(6)-Id*, and *aac(6')-Ib-cr* were designed in this study based on the sequence of the AMR genes predicted from the whole-genome sequencing of 10 isolates (data not shown). The information about the primers used is given in Table 1.

For the PCR, we used AmpliTaq® Gold^TM^ 360 Master Mix Kit (Applied Biosystems) in a total volume of 25 *µ*L, including 12.5 *µ*L Mix, 8.5 *µ*L water, 1 *µ*L enhancer, 1 *µ*L each primer (10 pmol/*µ*L), and 1 *µ*L DNA template (50–150 ng/*µ*L). Amplification reactions were carried out using a DNA Thermocycler (Eppendorf Mastercycler, Eppendorf-Netheler-Hinz GmbH, Hamburg, Germany) with suitable annealing temperatures shown in Table 1. We included positive and negative controls in each reaction. Immediately after electrophoresis in agarose gel (1.5%), the PCR products and DNA marker (100 bp, NEB) were viewed under UV transilluminator, and the gel image was documented.

## 3. Results

### 3.1. Bacterial Isolation and Phenotypic Characterization of AMR

From three different regions of the Emirate of Abu Dhabi, 165 *E. coli* were isolated from different animal species (camel = 42, sheep = 42, goat = 54, and poultry = 27).

**Figure 1:**
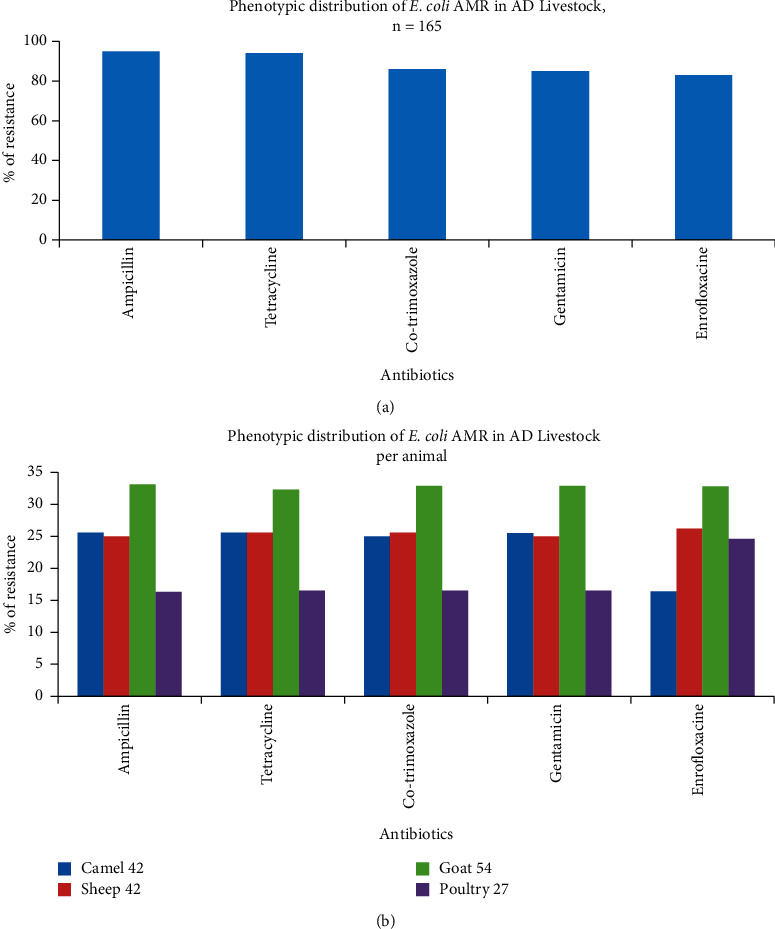
Antimicrobial resistance of *E. coli* isolates in the Emirate of Abu Dhabi livestock. (a) Per 165 *E. coli* isolates. (b) Per animal species (camel, sheep, goat, and poultry). A total number of animals are indicated.

Phenotypic analysis by the disk diffusion method showed that all the *E. coli* isolates are multidrug-resistant (MDR) since they showed resistance to 2, 3, 4, and even five classes of antibiotics by 5/165(3%), 17/165(10.3%), 47/165(28.5%), and 96/165 (58.2%), respectively (Table 2). Regardless of the source of *E. coli* isolates, the isolates were resistant to ampicillin, tetracycline, co-trimoxazole, gentamicin, and enrofloxacin by 157 (95.4%), 154 (93.6%), 141 (86%), 139 (85%), and 135 (82.7%), respectively (Figure 1(a)). All *E. coli* isolates from different animals were shown considerable resistance phenomena to all antibiotics tested (Figure 1(b)).

Furthermore, the patterns of “ampicillin-tetracycline-co-trimoxazole-gentamicin-enrofloxacin” (*n* = 96, 58.2%) and “ampicillin-tetracycline-co-trimoxazole-gentamicin” (*n* = 17, 10%) were the most frequent resistance profiles observed across all *E. coli* isolates (Table 2).

### 3.2. Detection of Antibiotic Resistance Genes by PCR

The PCR was used to test the presence or absence of the resistance genes to understand the molecular basis of the resistance observed in *E. coli*. The following AMR genes were targeted: *bla*_*CMY*_ and *blaTEM-1B* (for ampicillin); *tetA* and *tetB* (for tetracycline); *sul1*, *sul2*, and *sul3* (for sulfamethoxazole); *dfrA1* and *dfrA17* (for trimethoprim) (sulfamethoxazole and trimethoprim are components of co-trimoxazole); *aph(3”)-Ia*, *aph(6)-Id,* and aac(3)-IV (for gentamicin); and *qnrA* and *aac(6')-Ib-cr* (for fluoroquinolones).

**Figure 2:**
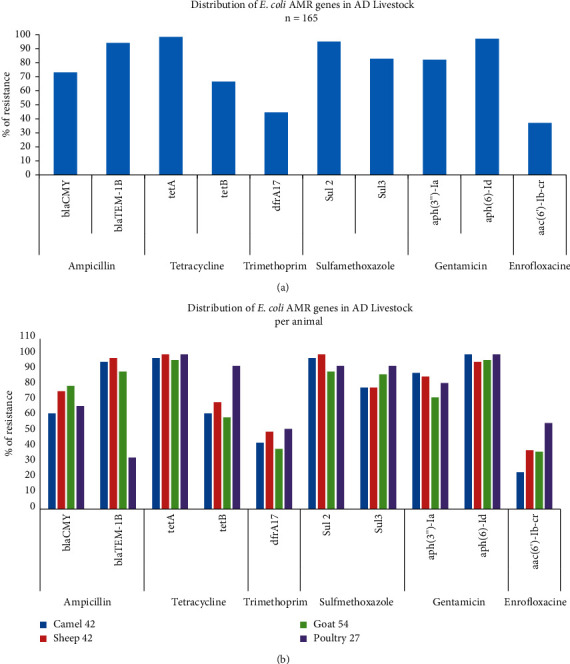
PCR detection of antimicrobial-resistant genes of *E. coli* isolated from livestock in the Emirate of Abu Dhabi. (a) Per 165 *E. coli* isolates and (b) per animal species (camel, sheep, goat, and poultry).

For ampicillin, *blaTEM-1B* showed a higher frequency (96.3%) than *bla*_*CMY*_ (72%) in the isolates (Figure 2(a)). About 50/160 (31.2%) isolates carry a single gene, either *bla*_*CMY*_ or *blaTEM-1B*, while 110/160 (68.8%) carry both genes. However, the *bla*_*SHV*_ gene was not detected.

For tetracycline, we detected the *tetA* and *tetB* in 162/164 (98.8%) and 112/164 (68.3%) of the isolates, respectively (Figure 2(a)). About 61/162 (37.7%) carry either *tetA* or *tetB,* and 101/162(62.3%) carry both genes.

Among the 164 cotrimoxazole-resistant *E. coli* isolates, the *dfra17, sul2*, and *sul3* genes were detected in 74 (44.5%), 156 (95%), and 138 (84%) isolates, respectively (Figure 2(a)). For sulfamethoxazole alone, one isolate 1/164 (0.6%) carried neither *sul2* nor *sul3*. Thirty-four (20.7%) isolates carried either *sul2* or *sul3*, while 130 (79.3%) carried both genes. Generally, in co-trimoxazole-resistant isolates, about 64/164 (39%) of the isolates carried the three genes (*dfra17, sul2*, and *sul3*). The genes *dfrA1* and *sul1* were not detected in our isolates.

The *aph(3”)-Ia* and *aph(6)-Id* genes were detected in 134 (82.1%) and 136 (98.2%) isolates, respectively, among the 164 gentamicin-resistant isolates (Figure 2(a)). Twenty-eight (17.1%) carried either *aph(3”)-Ia* or *aph(6)-Id* and 136 (82.9%) carried both genes. We could not detect *aac(3)-IV* gene in our isolates. All the 61 enrofloxacin-resistant isolates harbored the *aac(6')-Ib-cr* gene; however, *qnrA* gene was not detected. The detailed detection of the antibiotic resistance genes across different animals is shown in Figure 2(b).

### 3.3. Phenotypic: Genotypic Resistance Compatibility

Some *E. coli* isolates showed phenotypic resistance, but the resistance genes were not detected (mainly in gentamicin and enrofloxacin); the vice situation also exists. The overall phenotypic and genotypic compatibilities are 156/165 (94.5%), 153/165 (92.7%), 138/165 (83.6%), 137/165 (83%), and 73/165 (44.2%) for ampicillin, tetracycline, co-trimoxazole, gentamicin, and enrofloxacin, respectively, as detailed in Table 3.

## 4. Discussion

Currently, AMR is becoming a global concern requiring collaborated efforts in all fields, including humans, animals, and the environment, to identify its epidemiological patterns and apply proper approaches to improve treatment efficiency.

Epidemiological studies describing the dissemination of MDR in animals and the environment in the Middle East countries were conducted in only six out of the 15 countries [29]. In the present study, we analyzed the phenotypic and genotypic patterns of 165 MDR *E. coli* isolated from livestock in the Emirate of Abu Dhabi, UAE (based on samples received in the laboratory for bacterial investigation between 2014 and 2019) to provide an insight into this matter of concern. To the best of our knowledge, this is the first comprehensive study in this field.

Based on our results, all isolates 165/165 (100%) were phenotypically and genotypically multidrug-resistant (MDR) carry one or more resistant determinants (Tables 2 and 3). The most dominant antimicrobials with high resistance were ampicillin, tetracycline, and co-trimoxazole, and the lowest resistance is enrofloxacin, which is consistent with a previously published study in another region [30].

The genetic analysis in this study revealed that two AMR genes, namely, *blaTEM-1B* and *bla*_*CMY*_, are responsible for developing the resistance against ampicillin in the region. Similar results have been reported by other researchers [31, 32].

Generally, the resistance mechanisms for the tetracyclines fall in three categories: efflux pumps, ribosomal protection proteins (RPPs), or enzymatic inactivation. Over 40 different acquired tetracycline resistance determinants are recognized, that is, 38 *tet* (tetracycline resistance) [33]: 25 of the *tet* code for efflux pumps, 10 *tet* code for an RPP, and 3 *tet* genes for enzymatic inactivation mechanism. This study detected two tetracycline efflux genes *tetA* and *tetB* by 98.8% and 68.3%, respectively. Both determinants were among the most widespread *tet* genes found in enterobacteria [28, 34]. However, *tetA* marker is most common (67%) compared with *tetB* (31%) [8], consistent with our observations. By contrast, other studies have found that humans and ruminants carry less *tetA* and *tetB* [35].

For sulfamethoxazole (dihydropteroate synthetase)-resistant *E. coli,* it is generally attributed to the presence of *sul1*, *sul2*, and/or *sul3* genes [36], which are known to be associated with class 1 integrons [37]. In this study, we frequently detected *sul2* and *sul3* AMR genes. However, *sul2* was detected in 90% of the isolates, which is expected since it is the most frequent resistance mechanism to sulfamethoxazole in *E. coli* isolates [38, 39]. By contrast, other studies showed that *sul1*, *sul2*, and *sul3* have equal importance for sulfamethoxazole resistance in *E. coli* strains from food-producing animals in China [40]. Our PCR could not detect *sul1* gene, which indicates the absence or low abundance of class 1 integrons carry *sul1* gene [41] or even PCR failure, which needs to be investigated.

Trimethoprim inhibits the enzyme dihydrofolate reductase (DHFR) by competitively binding to its active site [42]. Acquired resistance to trimethoprim could be conferred by *dfrA* and *dfrB* gene families [43]. However, PCR only detected the *dfrA17* gene in 44.5% of the *E. coli* isolates. This distribution of the *dfrA17* gene could be attributed to their association with class 1 and class 2 integrons and plasmids [44, 45].

High rates of quinolones resistance (18.2%–92.5%) in *E. coli* from animal origins were observed worldwide [46], consistent with our phenotypic data (85% resistance). It is known that quinolone mediates resistance by four mechanisms: chromosome-encoded resistance, overexpression of naturally occurring efflux pumps, mutations of the molecular targets DNA gyrase and topoisomerase IV [47], and plasmid-mediated resistance genes, such as plasmids encodes a *qnr* determinant that protects DNA gyrase and type IV topoisomerase from quinolone inhibition [48] or plasmid encodes quinolone-resistant genes such as *aac(6')-Ib-cr* [49]. In the present study, we detected the *aac(6')-Ib-cr* gene in 37% of the resistant isolates indicating a plasmid-mediated determinant. However, this percentage of genes detected is less than phenotypic data, which suggests the involvement of other mechanisms since resistance to these drugs is derived from DNA gyrase mutations rather than by specific resistance genes and enzymes, which requires further studies.

The resistance phenotype and genotype were found not entirely consistent. Some isolates are phenotypically resistant, but no AMR genes were identified, and those are phenotypically susceptible but carrying resistance genes. For example, gentamicin genes were highly detected in the isolates (99.4%), while some strains were susceptible to most of the gentamicin that were tested (only 85% were resistant). In addition, the isolates were resistant to enrofloxacin (82.7%), while we detected only 37% of the determinants. The overall agreement between resistances is 94.5%, 92.7%, 83.6%, 83%, and 44.2% for ampicillin, tetracycline, co-trimoxazole, gentamicin, and enrofloxacin, respectively, as shown in Table 3. The apparent contradiction of susceptible isolates carrying resistance genes can be attributed to different possible reasons. The resistance genes may be unexpressed if they are distant from or associated with a weak promoter in an integron. Moreover, gene cassettes were known to confer resistance to most classes of antibiotics including that containing *β*-lactams, aminoglycosides, trimethoprim, and quinolones [50, 51]. However, gene cassettes are generally lack promoters and requires to be incorporated into the integron to be expressed (integron's promoter is required for the expression) [41, 52]. Thus, free gene cassettes (not integrated into an integron) are silent and confer no resistance [32]. Also, low minimum inhibitory concentration (MIC) test sensitivity provides an alternative explanation. Isolates could be falsely categorized as susceptible if the MIC breakpoint is higher than the resistance conveyed by the gene [43, 53]. By contrast, some resistant isolates did not carry any of the tested AMR genes, indicating other AMR genes (e.g., *tetD*, *tetE*, or *tetM* for tetracycline, and *bla*_*CMY*_ and *bla*_*TEM*_ for ampicillin) might be present, or other novel genetic resistant determinants exist [54]. Furthermore, factors such as enzymatic inactivation, target modification, or decreased outer membrane permeability may also be involved [55, 56]. Interestingly, at least one of the AMR genes was detected in most AMR in *E. coli* isolates (e.g., *tetA* or *tetB* for tetracycline) that encodes resistant phenotypes to the tested antibiotics in this study. Those isolates that were phenotypically resistant, but PCR negative should be screened for additional resistance determinants such as other possible genes, plasmid, transposons, and integrons.

Our data showed that *E. coli* from camel, sheep, goat, and poultry share a similar resistance gene pool for the different antibiotics tested. Regardless of animal species and number, the MDR pattern of *E. coli* has been observed in the three regions in which the isolated found resistant to all classes of antibiotics tested in this study. This resistance profile observed in the three regions of the Emirate of Abu Dhabi, possibly reflecting the same improper practice of antibiotic usage in animal husbandry. This study's result does not reflect a complete picture of *E. coli* AMR in the emirate of Abu Dhabi, because as mentioned early in this paper, the sample source of this study is the routine diagnostic samples received by our laboratory. Some limitations in our study have been observed. For example, only a few common resistance genes per antibiotic were investigated. Future studies in this region should consider additional genes for screening, especially for trimethoprim and enrofloxacin resistance, as these were poorly explained. Also, other animals like cattle should be included in our future screenings. The overall observed MDR *E. coli* isolated from livestock in the Emirate of Abu Dhabi, UAE, is far above previously reported figures of 20% in Finland, and it is similar to the 100% level reported for China [57].

Although the study did not include human samples, linking our results with previous human hospital-based data on *E. coli* AMR in the region is crucial. In UAE, in human, the *E. coli* was reported to have a high resistance to ampicillin in 1994 with the rates varying between 58 and 89%, and the resistance was increased for the two hospitals (Al-Ain and Tawam) in 2005. Similar resist pattern was observed for gentamycin and cotrimoxazole (Al-Ain Hospital) [19]. In another study, the extended-spectrum beta-lactamase (ESBL)-producing isolates found increased significantly over the period 2004–08 [21], and *E. coli* is one of the bacteria contributing to this phenomenon in UAE (from 7.0% to 22.3%) [21]. A third study in UAE has identified 53 (41%) of the 130 isolates as having ESBL phenotype; of these, 32 (60%) were *E. coli* [58]. Thus, understanding the interplay between humans and livestock in the context of AMR of *E. coli* based on a one-health approach is recommended.

In conclusion, the study generated preliminary data on the resistance of *E. coli* isolated from livestock in the Emirate of Abu Dhabi across five years and highlights the possible molecular mechanisms of the resistance. Furthermore, it suggests the improper use of antimicrobials in veterinary practice in the region. Thus, a comprehensive AMR survey system on a large scale should be initiated and *E. coli* could be one of the choices for the surveillance as other countries did [34, 59, 60]. Furthermore, the governmental agencies in UAE should critically supervise antimicrobial usage to reduce the threat to public health through the food chain and to avoid dissemination of antibiotic resistance in humans, animals, and the environment.

## Figures and Tables

**Table 1:** List of primers used in the study.

Antibiotic	Gene	DNA sequence (5'-3')	Annealing temperature (°C)	Product (bp)	Reference
Gentamicin	*aph(6)-Id*	F: ATCGTCAAGGGATTGAAACC R: GGATCGTAGAACATATTGGC	50	509	[25]
	*aph(3')-Ia*	F: ATGGGCTCGCGATAATGTCG R: AGAAAAACTCATCGAGCATC	60	734	[23]
	*aac(3)-IV*	F: CTTCAGGATGGCAAGTTGGT R: TCATCTCGTTCTCCGCTCAT	55	286	[28]
Ampicillin	*bla*_*SHV*_	F: TCGCCTGTGTATTATCTCCC R: CGCAGATAAATCACCACAATG	52	768	
	*blaCMY*	F: TGGCCAGAACTGACAGGCAAA R: TTTCTCCTGAACGTGGCTGGC	47	462	
	*blaTEM-1B*	F: TCCTTGAGAGTTTTCGCCCC R: TGACTCCCCGTCGTGTAGAT	60	634	This study
Sulfamethoxazole	*sul1*	F: TTCGGCATTCTGAATCTCAC R: ATGATCTAACCCTCGGTCTC	47	822	[28]
	*Sul2*	F: CACATTGCGGCGTTCTTTGA R: TTGCGGTTTCTTTTAGCGCC	60	241	This study
	*Sul3*	F: AGTGGGCGTTGTGGAAGAAA R: AGTAGCTGCACCAATTCGCT	60	361	
Trimethoprim	*dfrA1*	F: GGAGTGCCAAAGGTGAACAGC R: GAGGCGAAGTCTTGGGTAAAAAC	45	367	[27]
	*dfrA17*	F: GGCGTAATCGGTAGTGGTCC R: CCCCCGCCAGAGACATATAC	60	263	This study
Fluoroquinolone	*qnrA*	F: GGGTATGGATATTATTGATAAAG R: CTAATCCGGCAGCACTATTTA	50	670	[24]
	*aac(6')-Ib-cr*	F: GCGATGCTCTATGAGTGGCT R: TTTCTTCTTCCCACCGTCCG	60	220	This study
Tetracycline	*tet(A)*	F: GGTTCACTCGAACGACGTCA R: CTGTCCGACAAGTTGCATGA	57	577	[26]
	*tet(B)*	F: CCTCAGCTTCTCAACGCGTG R: GCACCTTGCTGATGACTCTT	56	634	

**Table 2:** The main phenotypic multidrug-resistant patterns of 165 *E. coli* strains were isolated from different livestock samples of the Emirate of Abu Dhabi.

No. of classes of drugs	Patterns of multidrug resistance (no. of isolates in this pattern)	No. of total isolates (%) (*n* = 165)
2	Ampicillin-enrofloxacin (*n* = 4)	5 (3%)
	Ampicillin-gentamicin (*n* = 1)	
3	Tetracycline-gentamicin-enrofloxacin (*n* = 4)	17 (10.3%)
	Tetracycline-co-trimoxazole-gentamicin (*n* = 1)	
	Ampicillin-tetracycline-gentamicin (*n* = 2)	
	Ampicillin-tetracycline-co-trimoxazole (*n* = 6)	
	Ampicillin-co-trimoxazole-gentamicin (*n* = 1)	
	Ampicillin-tetracycline-enrofloxacin (*n* = 2)	
	Ampicillin-gentamicin-enrofloxacin (*n* = 1)	
4	Tetracycline-co-trimoxazole-gentamicin-enrofloxacin (*n* = 2)	47 (28.5%)
	Ampicillin-co-trimoxazole-gentamicin-enrofloxacin (*n* = 3)	
	Ampicillin-tetracycline-gentamicin-enrofloxacin (*n* = 11)	
	Ampicillin-tetracycline-co-trimoxazole-enrofloxacin (*n* = 14)	
	Ampicillin-tetracycline-co-trimoxazole-gentamicin- (*n* = 17)	
5	Ampicillin-tetracycline-co-trimoxazole-gentamicin-enrofloxacin (*n* = 96)	96 (58.2%)

**Table 3:** Comparison of AMR in *E. coli* isolates according to phenotypic and genotypic testing.

Antibiotic	Phenotype	Genotype	% of dis-agreement	% of agreement
Ampicillin	P_−_ (*n* = 5)	G_+_ (*n* = 5)	9/165 (5.5%)	156/165 (94.5%)
	P_+_ (*n* = 4)	G_−_ (*n* = 4)		
Tetracycline	P_−_ (*n* = 11)	G_+_ (*n* = 11)	12/165 (7.3%)	153/165 (92.7%)
	P_+_ (*n* = 1)	G_−_ (*n* = 1)		
Co-trimoxazole	P_−_ (*n* = 26)	G_+_ (*n* = 26)	27/165 (16.4%)	138/165 (83.6%)
	P_+_ (*n* = 1)	G_−_ (*n* = 1)		
Gentamicin	P_−_ (*n* = 28)	G_+_ (*n* = 28)	28/165 (17%)	137/165 (83%)
Enrofloxacin	P_−_ (*n* = 10)	G_+_ (*n* = 10)	92/165 (55.8%)	73/165 (44.2%)
	P_+_ (*n* = 82)	G_−_ (*n* = 82)		

P_+/_G_−_ = Phenotypic resistant with no resistance gene identified. P_−/_G_+_ = Phenotypic susceptible with resistance gene identified.

## Data Availability

The data used to support the findings of this study are available from the corresponding author upon request.
